# Evaluating the Role of p38 MAPK in the Accelerated Cell Senescence of Werner Syndrome Fibroblasts

**DOI:** 10.3390/ph9020023

**Published:** 2016-04-28

**Authors:** Terence Davis, Amy J. C. Brook, Michal J. Rokicki, Mark C. Bagley, David Kipling

**Affiliations:** 1 Division of Cancer and Genetics, School of Medicine, Cardiff University, Heath Park, Cardiff, CF 14 4XN, UK; amy.brook@ymail.com (A.J.C.B.); rokickim@cardiff.ac.uk (M.J.R.); KiplingD@cardiff.ac.uk (D.K.); 2 Department of Chemistry, School of Life Sciences, University of Sussex, Falmer, Brighton, East Sussex BN1 9QJ, UK; m.c.bagley@sussex.ac.uk

**Keywords:** BIRB 796, cell ageing, fibroblasts, p38 MAP kinase, premature ageing, progeroid syndromes, SB203580, therapeutics, UR13756, VX-745

## Abstract

Progeroid syndromes show features of accelerated ageing and are used as models for human ageing, of which Werner syndrome (WS) is one of the most widely studied. WS fibroblasts show accelerated senescence that may result from p38 MAP kinase activation since it is prevented by the p38 inhibitor SB203580. Thus, small molecule inhibition of p38-signalling may be a therapeutic strategy for WS. To develop this approach issues such as the *in vivo* toxicity and kinase selectivity of existing p38 inhibitors need to be addressed, so as to strengthen the evidence that p38 itself plays a critical role in mediating the effect of SB203580, and to find an inhibitor suitable for *in vivo* use. In this work we used a panel of different p38 inhibitors selected for: (1) having been used successfully *in vivo* in either animal models or human clinical trials; (2) different modes of binding to p38; and (3) different off-target kinase specificity profiles, in order to critically address the role of p38 in the premature senescence seen in WS cells. Our findings confirmed the involvement of p38 in accelerated cell senescence and identified p38 inhibitors suitable for *in vivo* use in WS, with BIRB 796 the most effective.

## 1. Introduction

Werner Syndrome (WS) is a rare, autosomal recessive human disorder resulting from mutations in the *WRN* gene, which encodes the RECQ3 DNA helicase [1]. Individuals with WS show the premature onset of many clinical features of old age, including cataracts, skin atrophy, hair-greying and soft tissue calcification, together with age-related diseases such as type II diabetes, atherosclerosis and osteoporosis [2,3]. The median life expectancy is 47 years, with the major causes of death being myocardial infarction or mesenchymal neoplasms. With some exceptions, e.g., the absence of central nervous system degeneration, WS provides a stunning mimicry of normal ageing and is widely used as a model disease to investigate the mechanisms underlying normal human ageing [2,3].

Many, but not necessarily all, aspects of WS appear to be related to accelerated cell senescence. Cultured cells from normal individuals divide only a limited number of times before they enter a state of viable growth arrest termed cellular senescence, a condition that has been postulated to contribute to normal human ageing [2]. A key aspect of WS is that WS fibroblasts *in vitro* have a much reduced replicative capacity compared to normal fibroblasts [4]. This premature senescence has been postulated as a major contributor to the accelerated ageing of mitotic tissues *in vivo* in this syndrome [2]. WS thus provides an important model system to investigate the link between replicative senescence *in vitro* and normal ageing *in vivo*. 

Skin fibroblasts from individuals with WS grown *in vitro* show several characteristics of cells growing under conditions of stress, e.g., they have slow growth rates, an elongated cell cycle, and an altered morphology characterized by numerous F-actin stress fibres. In many respects, WS cells resemble fibroblasts that have undergone Stress-Induced Premature Senescence (SIPS) [5]. Of the many potential stressors that might be operative in WS cells, one that is particularly plausible is a DNA damage-like signal from the frequent stalled DNA replication forks that are a specific hallmark of cells deficient in RECQ3 [6]. This process has been termed DNA replication stress, and can activate cell cycle checkpoints resulting in cell cycle arrest [7]. 

SIPS is transduced in part by the p38α MAP kinase (MAPK14) signaling pathway [3], and young WS fibroblasts have elevated levels of activated p38 [5]. Treatment of WS fibroblasts with the p38α/β inhibitor SB203580 prevents the shortened replicative capacity, increases the growth rate, and alters the cellular morphology to resemble that seen for normal fibroblasts. This effect is associated with a down-regulation of p38 activation thus implicating p38 in these processes. Indeed, SB203580 appears to rescue all the *in vitro* accelerated ageing phenotypes of WS fibroblasts [5]. These data are consistent with the accelerated replicative senescence seen in WS cells resulting, at least in part, from activation of the p38 pathway; a SIPS-like state could then contribute to the accelerated ageing seen in WS individuals. Activation of p38α in WS would also be consistent with the high plasma levels of inflammatory cytokines such as tumor necrosis factor α (TNFα), and of inflammation-inducing cell surface molecules such as intercellular adhesion molecule-1 (ICAM-1), that are observed in WS individuals [8,9]. TNFα and ICAM-1 are known targets of p38α signaling and are associated with inflammatory conditions such as atherosclerosis [10,11]. This provides a plausible link between the underlying genetic lesion and the increased level of inflammatory diseases such as atherosclerosis and type II diabetes that are seen in WS [2]. This in turn raises the possibility of using p38α inhibitors, or interventions that target other components of this signaling pathway, as the basis for developing therapeutic approaches in this condition [12].

The bulk of the data on the role of p38 in WS has been based on the use of one inhibitor, SB203580 [3]. Whilst SB203580 has good selectivity against the α and β p38 isoforms and does not inhibit the γ and δ isoforms [13], it is not completely specific for p38α/β. For example, it has been shown to inhibit several other kinases with IC_50_ values similar to p38α/β, such as casein kinase 1 (CK1) isoforms (in particular CK1δ) and receptor-interacting serine-threonine kinase 2 (RIPK2) [14]. SB203580 also inhibits c-Jun-N-terminal kinase 2 (JNK2) and the kinase c-Raf1, albeit with significantly higher IC_50_ values than p38α/β. Of these, CK1 isoforms are involved in the transduction of the wingless (WNT)-signaling pathway that controls cellular proliferation [15], whereas JNK2 isoforms can cause growth arrest via stabilization of the cell cycle arrest protein p21^WAF1^ [16]. The RIPK2 kinase is upstream of p38α and can directly activate it, which makes the observation [5] that p38 is down-regulated upon SB203580 treatment in WS cells particularly intriguing. Since our previous work on accelerated senescence in WS fibroblasts was performed using SB203580 at 10 µM, it is formally possible that at least some of the effects seen with SB203580 are the result of inhibition of targets other than p38α. In addition, SB203580 does not provide a basis for *in vivo* studies due its toxicity profile [17]. 

This present study builds upon our initial observations with SB203580, and in particular seeks to assess the key issue of inhibitor selectivity by using novel p38 inhibitors with improved and/or different selectivity profiles, with a view to firmly establishing p38α as the effector in WS premature cellular senescence, and to find an inhibitor suitable for possible future *in vivo* use. The rationale behind the choice of inhibitors, including discussion of their modes of action, protein binding characteristics, kinase selectivity and toxicity profiles, is described in detail in [3].

## 2. Results

### 2.1. p38 Inhibitors Increase the Growth Rate of WS^tert^ Cells

**Figure 1:**
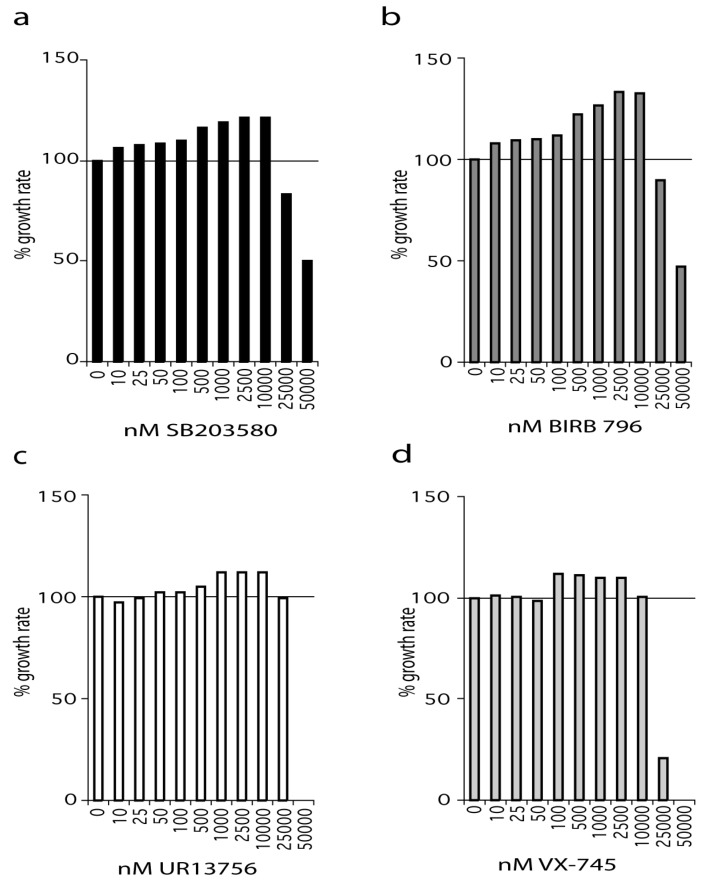
Effects of p38 inhibitors on the growth rate of WS^tert^ cells. Data for: (**a**) SB203580, (**b**) BIRB 796, (**c**) UR13756, (**d**) VX-745; (*n* = 1). For (**a**–**d**) the growth rate for each inhibitor concentration is expressed as a percentage of the DMSO control. The growth experiments for SB203580 and BIRB 796 titrations were repeated independently with the same overall result (see Supplementary Figure S1f, S1g). All growth rates were measured over a 42-day period.

WS^tert^ are WS fibroblasts that have been immortalized using human telomerase (see Materials and Methods) and were grown in standard medium supplemented with SB203580 at a final concentration ranging from 10 nM to 50 µM (Figure 1a). SB203580 treatment resulted in an increased growth rate compared to controls even at the lowest concentration used (10 nM). The effect on growth rate increased steadily with increasing SB203580 concentration, reaching a maximum between 2.5 µM and 10 µM. However, SB203580 maximally inhibited p38 at 500 nM, and has an IC_50_ for p38α of approximately 30 nM as measured by us, and others (Figure S1a) [18,19]. At 2.5 µM SB203580 resulted in an increase in growth rate of approximately 20% compared to DMSO controls. 

A similar situation was seen with BIRB 796, an inhibitor that binds to a novel allosteric binding site on p38α and has a reported IC_50_ of 18 nM [20]. We find that BIRB 796 is 100% effective at inhibiting p38 in the 2 h anisomycin test assay at 10 nM and above (Figure S1b). As with SB203580, BIRB 796 treatment resulted in an increased growth rate compared to controls even at the lowest concentration used (10 nM). Indeed, BIRB 796 showed a similar profile of effects to that seen for SB203580 over the entire concentration range, with the best growth rate increase being 30% at 2.5 µM (Figure 1b). BIRB 796 at 50 nM and above is fully active after 24 h in growth medium (Figure S1c).

UR13756 is an ATP competitive inhibitor that is highly specific for p38α/β with an IC_50_ of approximately 80 nM and maximal p38 inhibition above 500 nM in our test assay [21]. UR13756 had little effect on the growth of WS^tert^ cells up to 100 nM after which it was increasingly effective, flattening out between 1 µM and 10 µM, and thereafter becoming inhibitory (Figure 1c). UR13756 was fully p38 inhibitory after 24 h at 1 µM [21]. The maximal effect on growth rate (10%) was the same as seen with the lower SB203580 and BIRB 796 levels (Figure 1a,b).

VX-745 has an IC_50_ for p38α of approximately 30 nM [22]. When WS^tert^ cells were grown in increasing VX-745 concentration no effects were seen below 100 nM (Figure 1d). This concentration is above that required for 100% p38 inhibition in our 2 h test assay [22]. However, VX-745 was only 100% effective at inhibiting p38 at 1.0 µM and above after 24 h in growth medium [22]. Interestingly, as with UR13756, the maximum growth rate increase (10%) was the same as that seen using both SB203580 and BIRB 796 at levels lower than 500 nM.

### 2.2. The Effects of p53 Abrogation on the Growth Rate of WS^tert^ Cells

**Figure 2:**
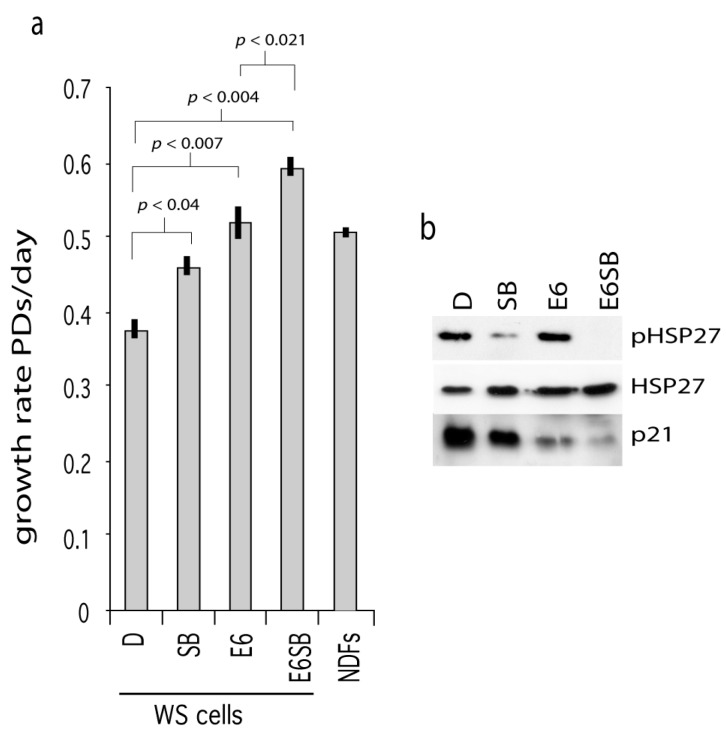
Effects of p53 abrogation on WS^tert^ cell growth. (**a**) Growth rates for WS^tert^ and WS^tert.E6^ cells, bars are ± SD (*n* = 2). D are DMSO treated cells, SB are cells treated with 2.5 µM SB203580, E6 are oncoprotein E6 expressing cells. NDFs are normal cells immortalized using human telomerase and grown in the presence of DMSO. Significance levels given for a subset of comparisons (*t*-test). (**b**) Immunoblot for the expression of p21^WAF1^, HSP27 and phosphorylated HSP27 (pHSP27) in WS^tert^ and WS^tert.E6^ cells.

One route by which p38 activity may result in cellular growth arrest is via activation of p53 or the stabilization of p21^WAF1^ [16,23]. To test this, WS^tert^ cells were transformed with the E6 oncoprotein (cells now termed WS^tert.E6^) to abrogate p53 function and then grown in the presence or absence of SB203580. E6 is a protein produced by human papilloma virus and binds to and degrades p53. As can be seen, the WS^tert.E6^ cells grew at a faster rate than control WS^tert^ cells (*p* < 0.007), and SB203580 treatment resulted in a further significant growth rate increases in both cases (Figure 2a).

The presence of E6 resulted in a down-regulation of p21^WAF1^ (Figure 2b; compare lane E6 with lane D) indicating successful abrogation of p53 function. SB203580 treatment resulted in p21^WAF1^ down-regulation in both WS^tert^ and WS^tert.E6^ cells (Figure 2b; compare lanes SB and E6SB with lanes D and E6 respectively). That the SB203580 is active in these cells is shown by its ability to reduce the levels of phosphorylated HSP27.

These data suggest that p38 and p53 can impinge on proliferation control in WS cells (possibly via activation of p21^WAF1^) by different routes as their abrogation or inhibition show synergy of action.

### 2.3. p38 Inhibition Increases the Replicative Capacity of Primary WS and Normal Fibroblasts

**Figure 3:**
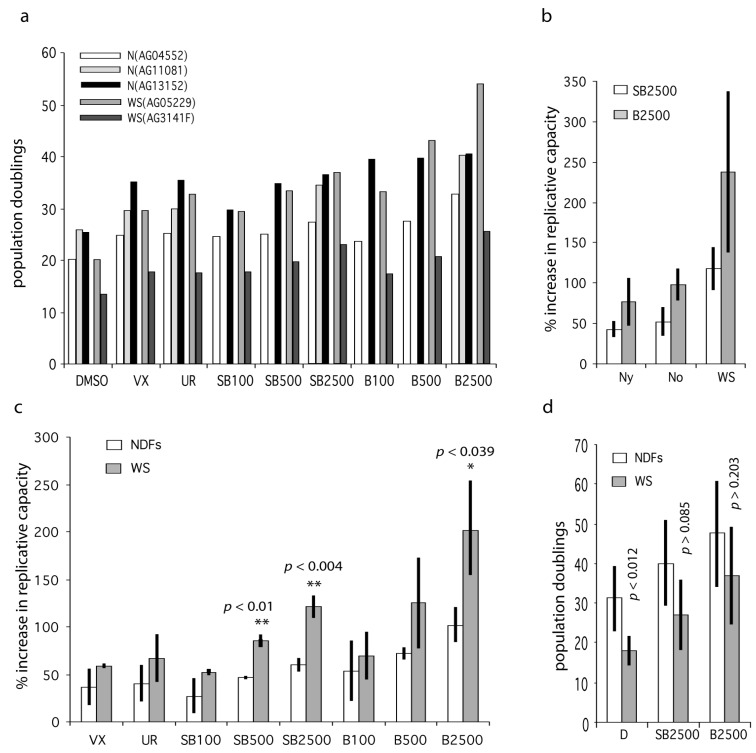
Effects of p38 inhibitors on the replicative capacity of WS and Normal primary fibroblasts. (**a**) Bar chart showing replicative capacity of fibroblasts from old and WS individuals. DMSO are controls, VX = 500 nM VX-745, UR = 1000 nM UR13756, SB = SB203580, B = BIRB 796; the numbers refer to nM concentrations, e.g., SB100 = SB203580 at 100 nM. (**b**) Data for the extended data set (Table S4) comparing NDFs from young (Ny) and old (No) individuals with WS. (**c**) Bar chart showing percentage increase in replicative capacity of fibroblasts from old and WS individuals expressed as mean ± SD (data from Table S3). (**d**) Mean replicative capacities (expressed as population doublings) of NDFs (all samples from Table S2) compared to WS fibroblasts ± inhibitor treatment. Statistical tests use the null hypothesis that the effects seen for inhibitors using WS fibroblasts are the same as that seen using NDFs, *t*-test. Significance levels only given for a subset of comparisons: ** highly significant (*p* < 0.01), * significant (*p* < 0.05).

We next assessed the effects of these inhibitors on the replicative capacity of primary WS fibroblasts and normal dermal fibroblasts (NDFs). The WS fibroblasts used were WS(AG05229) and WS(AG03141F), and the NDFs used were N(AG04552), N(AG1081) and N(AG13152). The growth conditions were the same as for the WS^tert^ cells. The donors of these NDFs were from elderly individuals (Table S1). We used VX-745 at 500 nM and UR13756 at 1.0 µM, as these were the lowest doses that gave a maximal growth response using the WS^tert^ cells (see Figure 1). For SB203580 and BIRB 796 we used three doses, 100 nM, 500 nM and 2.5 µM, so as to dissect in more detail the differential responses seen to these inhibitors. Due to their complexity all data from primary cells are presented in supplementary Tables S2 to S4, and only summary data are included in Figure 3.

The fibroblast strains were grown to replicative senescence at each inhibitor dose with the exception of N(AG11081), for which SB203580 and BIRB 796 were only used at 2.5 µM; the results are presented in Table S2, and plotted in Figure 3a. As can be seen the replicative capacities for fibroblasts from older individuals and WS are not greatly different. The ability of p38 inhibitors to enhance cellular growth were calculated as the percentage increase in experimental replicative capacity as compared to controls for each strain (Table S3). These latter were then averaged and the NDF strains were compared to the WS strains (Figure 3c). The reason for doing this is that the fibroblast strains had different experimental replicative capabilities due to different replicative histories, so measuring the percentage effect is a better measure of the responsiveness of the fibroblasts to p38 inhibition. For all inhibitor doses the two WS fibroblast strains are on average more responsive to p38 inhibitors than are NDFs. In addition, in only two situations (indicated in bold in Table S3) was any NDF more responsive to p38 inhibition than a WS culture. Inhibitors VX-745 and UR13756 were not as effective as the higher doses of SB203580 and BIRB 796, but were about as effective as the lower doses of SB203580 and BIRB 796. The comparisons that were statistically significantly different between WS fibroblasts and NDFs were SB203580 at 500 nM (*p* < 0.01) and 2.5 µM (*p* < 0.004), and BIRB 796 at 2.5 µM (*p* < 0.039).

We then extended the study to include several additional strains: three from WS, one from an elderly normal, one from a middle-aged normal, and three from younger normals (Table S1). The additional strains from normal individuals were used to explore whether the response to p38 inhibition was limited to fibroblasts from elderly and Werner syndrome donors. All strains were grown to replicative senescence as before, using SB203580 and BIRB 796 at 2.5 µM, this being the inhibitor concentrations at which we see the maximal differences between NDFs and WS fibroblasts. The data are summarized in Tables S2 and S4 and Figure 3b. The first thing of note is that cells from old individuals were not significantly more responsive to p38 inhibitors than cells from young individuals, or to cells from a middle-aged donor. However, WS cells were significantly more responsive than NDFs to both SB203580 and BIRB 796, not only when compared to all the NDFs used (*p* < 0.00014 and *p* < 0.0019 respectively), but also when compared to the cohorts of fibroblasts from the young and old individuals (Table S4). 

These increases were also significant when the actual replicative capacities of the various fibroblasts strains were analyzed (Figure 3d). The mean replicative capacity of the WS strains was significantly reduced when compared to the eight NDF strains (18.0 ± 3.6 CPDL compared to 31.1 ± 8.0 CPDL; *p* < 0.012: Table S2), in agreement with the reported data for WS strains [4,24]. Treatment with both SB2303580 and BIRB 796 increased the replicative capacity of WS fibroblasts to within the range seen for inhibitor-treated NDFs (Figure 3d; Table S2), with BIRB 796 more effective than SB203580. This was seen particularly for the strain WS(AG05229) where BIRB 796 increased the replicative capacity from 20.9 to 53.7 CPDL, a value that is greater than seen in six of the BIRB 796 treated NDF strains (Table S2). Finally, for strain WS(AG12800) the replicative capacity was increased by 490% and 865% using SB203580 and BIRB 796 respectively, with the latter increasing the replicative capacity from 2 CPDL to 19.3 CPDL (Table S2, and Figure S1d).

### 2.4. p38 Inhibitors Prevent the Stressed Cellular Morphology of Primary WS Fibroblasts

**Figure 4:**
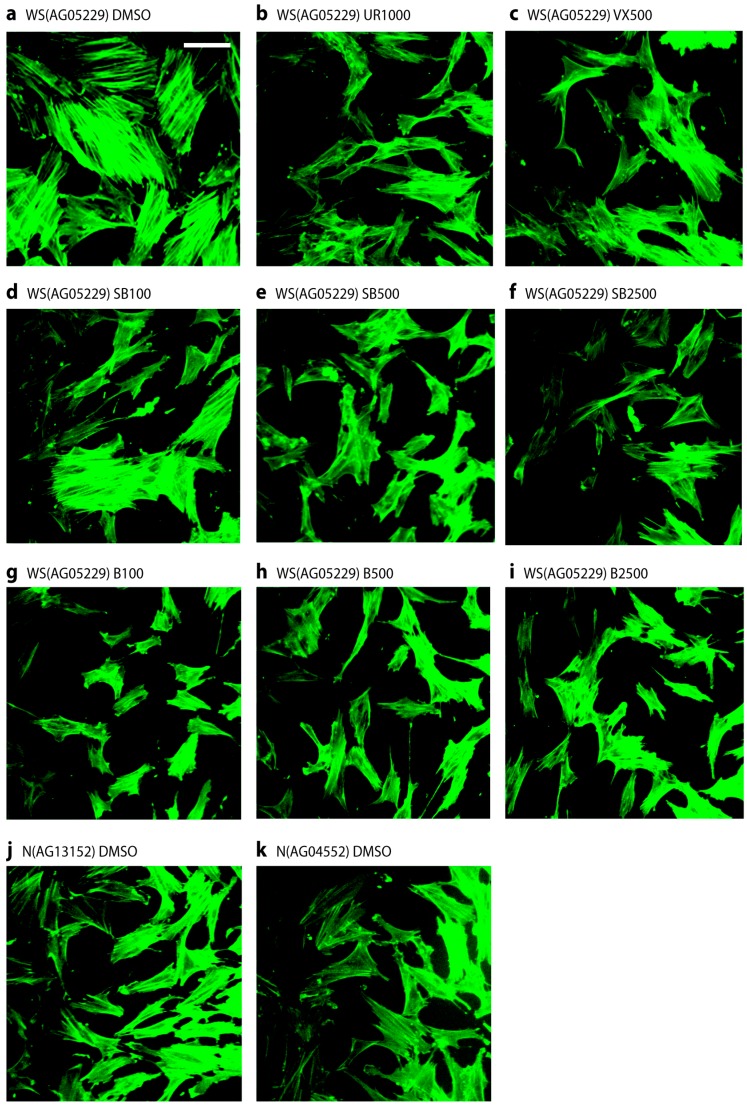
Stress fibre phenotypes of WS(AG05229) cells. Phalloidin stains for WS(AG05229) cells (**a**–**i**) and NDFs (**j** and **k**). Each panel labeled with strain and inhibitor used (symbols for inhibitors as in legend to Figure 3). These are representative figures from many experiments. Bar = 100 µm for each panel as all are at the same scale. *Note:* A copy of this figure is also provided as Figure S2 and is available online.

One of the principle phenotypic characteristics of primary WS cells is that, even at low population doubling levels, they display an altered morphology with many of the cells resembling senesced cells, in that they are enlarged and granular with numerous F-actin stress fibres visible [5]. This is illustrated for strain WS(AG05229) in Figure 4a when the cells were treated with fluoresceinisothiocyanate labeled phalloidin to stain filamentous actin, which clearly visualizes the presence of numerous strongly stained fibres. Treatment of these cells with p38 inhibitors largely prevented the development of these actin stress fibres, resulting in cells with a smaller and more regular morphology (Figure 4b–i). It is clear from Figure 4d that SB203580 at 100 nM was less effective, which was probably due to less than 100% p38 inhibition at this inhibitor concentration (Figure S1a). Similar results were seen for the WS strain WS(AG03141F) (not shown). For comparison, two NDF strains are shown that lack this stress fibre phenotype (Figure 4j,k), these being typical of the NDF strains used. Inhibitor treatment had little effect on the morphology of NDFs (not shown).

## 3. Discussion

The rationale behind this study is threefold. First, our use to date of a single p38 inhibitor (SB203580) is problematic due to its known selectivity issues [19]; thus, it remained unclear whether p38α was the main kinase involved in premature cell senescence in WS. Second, SB203580 has toxicity issues [17] that preclude long term *in vivo* use, motivating the study of other inhibitors that might be clinically useful. Third, we wished to dissect in more detail any differences in sensitivity of fibroblasts from different age groups to p38 inhibition.

SB203580 is known to inhibit, at least *in vitro*, several other kinases that have involvement in the control of cellular proliferation, namely CK1δ, c-Raf-1, and the various JNKs, in addition to various other kinases with less well-characterized roles such as RIPK2 [14,19]. The various p38 inhibitors chosen for this study were synthesized (see [3] for details) and tested for their effects on WS cells. These inhibitors were of different potencies, were chemically distinct and showed a different (known) range of off-target selectivities (discussed in [3,25]). To optimize the use of limited primary WS cell material we first tested these inhibitors on a telomerase-immortalized WS cell tester strain (WS^tert^) that retains the slow growth and stressed morphology seen in primary WS cells [26].

All of the p38 inhibitors used had a positive effect on the growth rate of WS^tert^ cells, although each was different. The growth rate of WS^tert^ cells showed a relatively muted response to the inhibitors VX-745 and UR13756, although this did occur at doses consistent with p38 inhibition. These are ATP competitive inhibitors with high specificity for the p38α and p38β kinases, and do not inhibit the JNKs [27,28] that are in the same family of stress-induced MAP kinases. A larger effect on the growth rate of the WS^tert^ cells was seen for the kinase inhibitors SB203580 and BIRB 769, again at doses consistent with p38 inhibition. The effect was approximately twice that seen for VX-745 and UR13756. SB203580 is an ATP competitor with high specificity for the p38α and p38β kinases and is known not to significantly inhibit the JNKs in cells at the concentrations at which it is most effective [21,29]. Finally, BIRB 796 was most effective at increasing the growth rate of WS^tert^ cells and it was the most potent of all the inhibitors at p38 inhibition. This inhibitor has a different binding mode to the others and acts both by binding to an allosteric site and as an ATP competitor, and can block p38 activation as well as its activity [30], attributes that may explain its greater potency. The growth rate increases found when using these inhibitors, although small, are highly reproducible. In addition to increasing growth rate, these inhibitors also alleviated the stress-fibre phenotype of the WS^tert^ cells (not shown), a phenotype known to be the result of p38 activation [31,32]. Finally, all inhibitors reduce cellular growth rates at high concentrations possibly due to non-specific cell toxicity issues at these doses.

With regard to kinases CK1δ, c-Raf1, the JNKs and RIPK2, the data reported in this paper are supported by observations that inhibition of CK1δ results in complete cessation of growth and increased apoptosis in WS cells [33], a result not inconsistent with the known biology of CK1δ [34]. Thus, if SB203580 inhibits CK1δ *in vivo*, cell cycle arrest should occur rather than an extension of replicative capability. In addition, the p38 inhibitors VX-745 and BIRB 796 do not bind to or inhibit CK1δ [13,14,28], and both result in an increased replicative capacity in WS cells.

Both SB203580 and BIRB 796 can inhibit c-Raf1 [35,36]. However, it has been noted that c-Raf1 acts in a negative feedback loop via autophosphorylation, and its inhibition results in c-Raf1 hyper-activation [36]. In addition, *in vivo* inhibition using various c-Raf1-specific inhibitors led to up-regulation of compensatory pathways that left its downstream pathways active [33]. These data suggest that if SB203580 does inhibit c-Raf1 *in vivo*, such a compensatory effect would be activated, leading to little effect on growth. That both VX-745 and UR13756 also result in growth enhancement, albeit to a lesser extent than SB203580, with neither inhibitor affecting c-Raf1 [27,28], provides support for this, as does the observation that a potent c-Raf1 specific inhibitor had no effect on WS cell growth [33].

SB203580 has been reported to inhibit the stress-associated JNKs in *in vitro* assays, albeit with an IC_50_ value of between 0.7 µM and 11 µM [18,19]. However, total JNK activity is only partially inhibited by SB203580 *in vivo* at concentrations above 10 µM or by BIRB 796 at 2.5 µM and above, and is not inhibited by UR13756 and VX-745 [13,21,27,28] (see also Figure S1e showing no inhibition of JNK1/2 by either SB203580 or BIRB 796 at the concentrations used in this work compared to the effect of a JNK inhibitor [37]). These data, and recent evidence using specific JNK inhibitors which shows that complete inhibition of JNK has no effect upon replicative capacity in WS cells [37], strongly suggest that these effects are not due to JNK activity.

The kinase RIPK2 is inhibited by SB203580 at lower doses than needed to inhibit p38α, and its inhibition suppresses p38α activation [38]. This is interesting as treatment of WS cells with SB203580 suppresses p38 activation [5]. Previous work using RNA interference suggested that RIPK2 was not involved in WS cell growth [33], although with the caveat that RNA knockdown is not the same as small molecule inhibition of a kinase. The data presented here using several p38 inhibitors provides further evidence that RIPK2 is not involved, as the inhibitors VX-745 and BIRB 796 do not bind to or inhibit RIPK2 [14,28].

Having established that the increased growth rate of WS^tert^ cells using SB203580 results from inhibition of p38α this study then tested the effects of p38 inhibition on primary WS fibroblasts in comparison to a panel of NDFs. As with the WS^tert^ cells, treatment of WS fibroblasts with all the inhibitors used (VX-745, UR13756, SB202580 and BIRB 796) resulted in increases in replicative capacity. These increases showed the same pattern in primary cells as seen in the WS^tert^ cells, with SB203580 and BIRB 796 being more potent. However, even though the effects were consistently greater for WS cells than for NDFs, this was only significant for the highest doses of SB203580 and BIRB 796. For the latter inhibitors, this was true when WS cells were compared to NDFs by either a panel of young individuals or a panel of old individuals (Figure 3b). The increases were also significant for SB203580 and BIRB 796 when measured as population doublings achieved (CPDL), and resulted in an increase in the replicative capacity of WS cells from below that seen in NDFs to within the normal NDF range (Figure 3d). These data support the hypothesis that the shortened replicative capacity seen in primary WS fibroblasts is due in large part to p38-dependent SIPS. 

The effects of the inhibitors SB203580 and BIRB 796 were essentially the same for the fibroblasts from old and young individuals, suggesting that p38-dependent SIPS is not occurring to a higher level in cells from older individuals, which fits with the evidence that fibroblasts from older individuals do not have a significantly reduced replicative capacity compared to fibroblasts from younger individuals [39]. These data also strongly suggest that WS fibroblasts are not simply a ‘phenocopy’ of cells from an old individual, and provide support for the concept of SIPS leading to a reduced capability of WS cells to undergo proliferation as a result of p38α activation, probably due to ongoing DNA replication stress [7]. This replication stress manifests as a difficulty in replicating certain DNA sequences, such as the common fragile sites (CFSs) and telomeres, and WS fibroblasts show elevated telomeric truncations and expression of CFSs [40,41].

This concept raises the question as to how inhibiting the p38-dependent SIPS leads to an increase in the replicative capacity of WS fibroblasts, since DNA replication stress should still be occurring. It is possible that by preventing SIPS, a second mechanism is activated to process the replication stress to a successful resolution and thus allow cell division, and in this regard it is interesting to note that p38 activation is down-regulated in SB203580 treated cells [5], suggesting that the stress is alleviated. Recent evidence has demonstrated that in the absence of the RECQ3 helicase, a second pathway is indeed activated in WS cells, one that involves activation of the MUS81 endonuclease and the RECQL5 and RECQ2 helicases [42,43,44], and increased RECQL5 expression can overcome replication stress in human cells [45]. It is possible that in WS activation of p38 leading to SIPS is a rapid process to protect cells from excessive DNA damage, with the secondary back-up pathways being slow acting. When p38 is inhibited this then allows the back-up pathways to operate leading to resolution of the replication stress and progression though the cell cycle. This is, of course, speculative, but evidence is increasing to support this viewpoint, although these back-up pathways are not fully understood [7,43].

Our data suggest that the most potent inhibitor is BIRB 796. As all of these inhibitors target p38α, some with potencies in *in vitro* assays almost as high as BIRB 796, why is the latter more effective? It is possible that BIRB 796 is simply more stable in the growth media during these *in vitro* studies, a possibility supported by the reduced potency of inhibitors such as VX-745 seen in the 24 h assays and over longer time periods [22]. A second possibility is that this is due to BIRB 796 having a different binding mode from the other inhibitors, since it binds not only to the ATP-binding pocket but also to an adjacent allosteric site; thus it binds more tightly to p38α and is not readily displaced by ATP competition [20]. A third possibility relates to the altered binding mode as BIRB 796 can inhibit all four p38 isoforms (unlike the other inhibitors that only target p38α and p38β) [13], and recent data suggest that premature cellular senescence can result from activation of p38γ and p38δ [46,47]. As p38γ causes cell cycle arrest by phosphorylation of p53 thus up-regulating p21^WAF1^, whereas p38α stabilizes p21^WAF1^ directly and appears not to phosphorylate p53 [16,46,47], the observation that abrogation of p53 by E6 and p38α inhibition by SB203580 show a synergistic effect on cellular growth rates and p21^WAF1^ levels suggest the possibility that both p38α and p38γ may be involved in the premature senescence seen in WS cell that then may explain the increased potency of BIRB 796. As BIRB 796 has been used extensively in clinical trials [3], this inhibitor may be useful for future *in vivo* studies, such as in the existing WS mouse model [48] to test the idea that SIPS in WS does indeed lead to the accelerated ageing in this syndrome. In addition, as accelerated ageing in WS is associated with increased inflammatory conditions and senescent cells produce inflammatory cytokines, the observation that BIRB 796 suppresses this cytokine production [49] suggests that, if SIPS is operating in WS, then p38 inhibitors may have clinical utility to alleviate these inflammatory conditions.

Finally, even though the inhibitors VX-745 and UR13756 are less effective than BIRB 796, they do reduce the accelerated senescence seen in WS cells and alleviate the stressed cellular morphology. Also, as VX-745 is tolerated in mouse for up to six months [3] this inhibitor may be useful for proof-of-principle *in vivo* studies in the mouse WS model [48]. In addition, as this work confirms a role for p38 isoforms in premature cellular senescence, it can underpin future *in vivo* studies for p38 inhibitors that are still at the developmental stage that may have improved clinical applicability in humans.

## 4. Materials and Methods 

### 4.1. Protein Kinase Inhibitors

The protein kinase inhibitor SB203580 was obtained from Tocris Chemical Co. (Bristol, UK). The kinase inhibitor BIRB 796 was synthesized according to [30]. VX-745 was synthesized according to [22]. UR13756 was synthesized according to [21]. The synthesis routes, modes of action and protein binding characteristics for these inhibitors are described in detail in [3].

### 4.2. Cells and Cell Growth

Adult dermal primary fibroblasts were obtained from the Coriell Cell Repositories (Camden, NJ, USA) and are listed in Table S1. All cell strains have been given a prefix to indicate their group followed by the Coriell strain identifier in parentheses; for example, N(AG04552) is the AG04552 strain of normal dermal fibroblasts (NDFs). Primary NDFs were chosen from a range of elderly and younger individuals with a range of replicative capabilities. WS^tert^ are WS(AG03141) fibroblasts that have been immortalized by the ectopic expression of human telomerase and have been described previously [26]. The human telomerase immortalized HCA2^tert^ cells have been described previously [22].

Due to limited supplies of primary WS fibroblasts, initial growth experiments to determine the effects of novel kinase inhibitors on cellular growth were performed using WS^tert^ cells. Despite being immortal, these telomerized cells retain the slow growth rate typical of primary WS cells and show a similar response to SB203580 treatment [26]. Thus, these cells can be used to test the effects of modulators of the p38-signalling pathway prior to their use on primary cells.

Cells were grown in DMEM as previously described [50]. Population doublings (PDs) achieved at each cell passage were calculated according to the formula: PDs = log(*N*_t_/*N*_o_)/log2, where *N*_t_ is number of cells counted and *N*_o_ is number of cells seeded. The number of PDs at each passage was then totaled to give a cumulated population doubling level (CPDL). For experiments using immortalized cells the proliferation rate was calculated by dividing the CPDL by the number of days of the experiment concerned and expressed as PDs/day. For primary cells the CPDL is quoted in the Supplementary Tables. For assessing the effects of the various kinase inhibitors the culture medium was supplemented with the inhibitor dissolved in DMSO, with the medium being replaced daily unless otherwise stated. For controls an equivalent volume of the inhibitor solvent (DMSO) was added to the medium.

### 4.3. Determination of the Ability of Inhibitors to Inhibit the p38 Pathway

The ability of individual compounds to inhibit the p38 stress-signaling pathway was tested in anisomycin-treated HCA2^tert^ cells using an ELISA-based readout (Cell Signalling, NEB, Hitchin, UK) as described in [51]. Kinase activity was detected using antibodies specific for the phosphorylated forms of HSP27 and antibodies that detect total levels of HSP27, the degree of activation being recorded as the ratio of phospho-protein/total protein; the data for SB203580 and BIRB 796 are given in Figure S1a,b. Data for the other inhibitors has been published previously [21,22].

To test the efficacy of these inhibitors under experimental growth conditions, the pre-incubation time with selected inhibitor concentrations was increased to 24 h to match the daily feeding regimen of the growth experiments; the assay was otherwise identical (see Figure S1c).

### 4.4. Immunofluorescence Microscopy

Actin staining for immunofluorescence microscopy was performed essentially as described [5].

### 4.5. Immunoblot Analysis

All procedures for the immunoblot analyses were carried out as described [5]. Antibodies used were: mouse monoclonal anti-HSP27 (G31), rabbit polyclonal anti-phospho(S82)-HSP27, rabbit polyclonal anti phospho(T183/Y185)-JNK1/2, rabbit monoclonal anti-JNK1/2 (56G8), rabbit monoclonal anti-c-Jun (60A8) (Cell Signalling, New England Biolabs, Hitchin, UK) and mouse monoclonal anti-p21^WAF1^ (6B6; Becton Dickinson, Swindon, UK).

### 4.6. Retroviral Gene Transfer

Amphotropic retrovirus vectors expressing the HPV16 E6 oncoprotein were constructed and gene transfer was carried out as described previously [52].

## 5. Conclusions

Four p38 inhibitors (SB203580, BIRB 796, VX-745 and UR13756) have been used to test the role of p38α MAPK in the premature senescence seen in primary fibroblasts from the premature ageing Werner syndrome. All four successfully reduced the accelerated cell senescence to some degree, although BIRB 796 was by far the most potent, and its use extended the replicative capacity of WS cells to within the range seen for fibroblasts from normal individuals. As these inhibitors all have different protein binding modes and specificity profiles (their only common target being p38), these data essentially confirm that premature WS cell senescence is due to activation of p38. Also, as some of these inhibitors are tolerated in the mouse for up to six months, they may be useful for future proof-of-principle *in vivo* studies to try to establish a link between *in vitro* premature cell senescence and *in vivo* premature ageing in the mouse model of Werner syndrome. This in turn may underpin future *in vivo* human studies into whether p38 inhibitors may have clinical potential in human ageing.

## Supplementary Materials

The following are available online at http://www.mdpi.com/1424-8247/9/2/23/s1, Figure S1: Supplementary figures, Figure S2: Figure 4, Table S1: fibroblast strains used in this study, Table S2: Comparison of CPDL for inhibitor treated normal and WS fibroblasts, Table S3: Percentage increases in replicative capacity for primary fibroblasts using p38 inhibitors, Table S4: Percentage increases in replicative capacity using SB203580 and BIRB796.
