# Supplementary material for: Evaluating the Role of p38 MAPK in the Accelerated Cell Senescence of Werner Syndrome Fibroblasts
Source: Pharmaceuticals (Basel). 2016 Apr 28;9(2):23. doi: 10.3390/ph9020023 (PMC4932541; doi:10.3390/ph9020023)
Supplement: Supplementary file 1 [file pharmaceuticals-09-00023-s001.pdf]

Supplementary Materials: Evaluating the Role of p38 MAPK in the Accelerated Cell Senescence of Werner Syndrome Fibroblasts

Terence Davis, Amy J.C. Brook, Michal J. Rokicki, Mark C. Bagley and David Kipling

Table S1. Fibroblasts strains used in this study.

Strain [a]	Age of Donor	Replicative History [b]
	**Normal (N)**	
N(GM05399A)	1	16 PDs
N(GM05400A)	6	15 PDs
N(AG06234)	17	15 PDs
N(AG13156)	44	9 PDs
N(AG04552)	65	10 PDs
N(AG11081)	78	13 PDs
N(AG13152)	79	10 PDs
N(AG11020)	79	10 PDs
	**Werner Syndrome (WS)**	
WS(AG05229)	25	7 PDs
WS(AG03141B) [c]	30	12 PDs
WS(AG03141D) [c]	30	14 PDs
WS(AG03141F) [c]	30	6 PDs
WS(AG12800)	25	Passage 1

[a] To avoid confusion when referring to cell strains in this paper a prefix has been added to the strain code; [b] Number of population doublings (PDs) achieved at the Coriell Cell Banks prior to the receipt of the cells and the start of these growth experiments (where a passage number is given the PD value used has been set as zero); [c] Different batches of AG03141 WS cells.

Table S2. Comparison of CPDL [a] for inhibitor treated normal and WS fibroblasts.

Strain	PD at Start	PDs Achieved								
		Cont [b]	VX	UR	SB100	SB500	SB2500	B100	B500	B2500
					**Normal (N) young individuals (<25 years)**					
N(AG06234)	15	34.1	nd [c]	nd	nd	nd	40.7	nd	nd	42.8
N(GM05399A)	15	41.6	nd	nd	nd	nd	55.5	nd	nd	68.6
N(GM05400B)	16	43.4	nd	nd	nd	nd	54.7	nd	nd	66.0
Mean ± SD [d]		39.7 ± 4.9					50.3 ± 8.3			59.1 ± 14.2
					**Normal (N) old individuals (>64 years)**					
N(AG04552)	10	20.3	24.7	25.1	24.3	25	27.1	23.5	27.3	32.6
N(AG11020)	10	26.7	nd	nd	nd	nd	30.8	nd	nd	40.8
N(AG11081)	13	26.7	29.2	29.7	nd	nd	34.1	nd	nd	40.3
N(AG13152)	10	26.5	35.0	35.6	28.9	34.2	36.5	38.8	39	40.6
Mean ± SD[d]		25.1 ± 3.17					32.1 ± 4.1			38.6 ± 3.98
					**Normal (N) 44 years old individual**					
N(AG13156)	9	29.9	nd	nd	nd	nd	41.6	nd	nd	48.6
Mean ± SD [e]		31.14 ± 8.0					40.1 ± 10.4			47.5 ± 13.0
					**Werner syndrome (WS)**					
WS(AG05229)	7	20.9	29.2	32.6	28.3	33.2	36.7	32.9	42.9	53.7
WS(AG03141B)	12	16.9	nd	nd	nd	nd	21.5	nd	nd	36.1
WS(AG03141D)	14	20.7	nd	nd	nd	nd	nd	nd	nd	32.3
WS(AG03141F)	6	13.4	17.7	17.1	17.1	19.4	22.9	17.3	20.2	25.8
Mean ± SD [d]		18.0 ± 3.6					27.1 ± 8.4			36.9 ± 11.9
p (t-test) WS v N (all)		<0.012					>0.085			>0.203
WS(AG12800)	0	2.0	nd	nd	nd	nd	11.8	nd	nd	19.3

[a] CPDL = cumulative population doubling level (see methods for explanation of term); [b] Cont = DMSO treated cells, VX = 500 nM VX-745, UR = 1000 nM UR13756, SB = SB203580, B = BIRB 796. The numbers refer to nM concentrations, e.g., SB100 = SB203580 at 100 nM; [c] nd = not done; [d] Mean ± SD. Result only given for DMSO, SB2500 and B2500; [e] refers to all eight NDFs.

Table S3. Percentage increases in replicative capacity for primary fibroblasts using p38 inhibitors [a].

Strain	Inhibitors [b]							
	**VX**	**UR**	**SB100**	**SB500**	**SB2500**	**B100**	**B500**	**B2500**
**N (Normal)**								
N(AG04552)	42.7	45.6	38.5	45.6	66	31.5	68.5	119
N(AG11081)	18.2	22.0	nd [c]	nd	54	nd	nd	99.3
N(AG13152)	51.5	**55.1**	14.5	46.7	60.6	**74.5**	75.7	85.4
Mean ± SD	37.5	40.9	26.5	46.1	60.2	53	72.1	101.2
	17.2	17.1	17.0	0.78	6.0	30.4	5.1	16.9
**WS (Werner Syndrome)**								
WS(AG05229)	59.7	84.2	53	88.5	114	86.3	158.3	236
WS(AG03141F)	58.1	50	50	81.1	128.4	52.8	91.9	168
Mean ± SD	*58.9*	*67.1*	*51.5*	*84.8*	*121.2*	*69.6*	*125.1*	*201.8*
	1.13	24.2	2.1	5.23	10.2	23.7	46.9	48.4
p (t-test)	>0.19	>0.24	>0.17	<0.01	<0.004	>0.6	>0.25	<0.039

[a] The % increase is determined with reference to starting PD as far as possible: e.g., for N(AG04552) using SB203580 at 2500 nM the replicative capacity increase is (27.1 PDs–10 PDs)/(20.3 PDs–10 PDs) = 1.66% or a 66% increase in experimental replicative capacity. See Table S1 for starting PDs; [b] For definitions see Table S2; [c] nd = not done.

Table S4. Percentage increases in replicative capacity using SB203580 and BIRB796 [a].

**Strain** [b]	**Inhibitors** [c]	**SB2500**	**B2500**
Ny (Normal) < 25 years			
N(AG06234)		34.5	45.6
N(GM05399A)		52.2	101.5
N(GM05400B)		41.3	82.5
Mean ± SD		42.7 ± 8.9	76.5 ± 28.4
N (Normal) 44 years			
N(AG13156)		56.0	89.5
No (Normal) > 64 years			
N(AG04552)		66	119
N(AG11020)		24.6	84.4
N(AG11081)		54	99.3
N(AG13152)		60.6	85.4
Mean ± SD		51.3 ± 18.5	97.1 ± 16.1
p (t-test) Ny v No		>0.49	>0.27
WS (Werner Syndrome)			
WS(AG05229)		114	236
WS(AG03141F)		128.4	168
WS(AG03141B)		93.9	392
WS(AG03141D)		nd [d]	173
Mean ± SD		112 ± 17.3	242.1 ± 104.6
p (t-test) WS v N (all)		<0.00014	<0.0019
p (t-test) WS v Ny		<0.0036	<0.048
p (t-test) WS v No		<0.007	<0.034
WS(AG12800)		490	865

[a] For details see Table S2 notes; [b] Ny = young individuals, No = old individuals; [c] Inhibitors at 2.5 μM; [d] nd = not done.

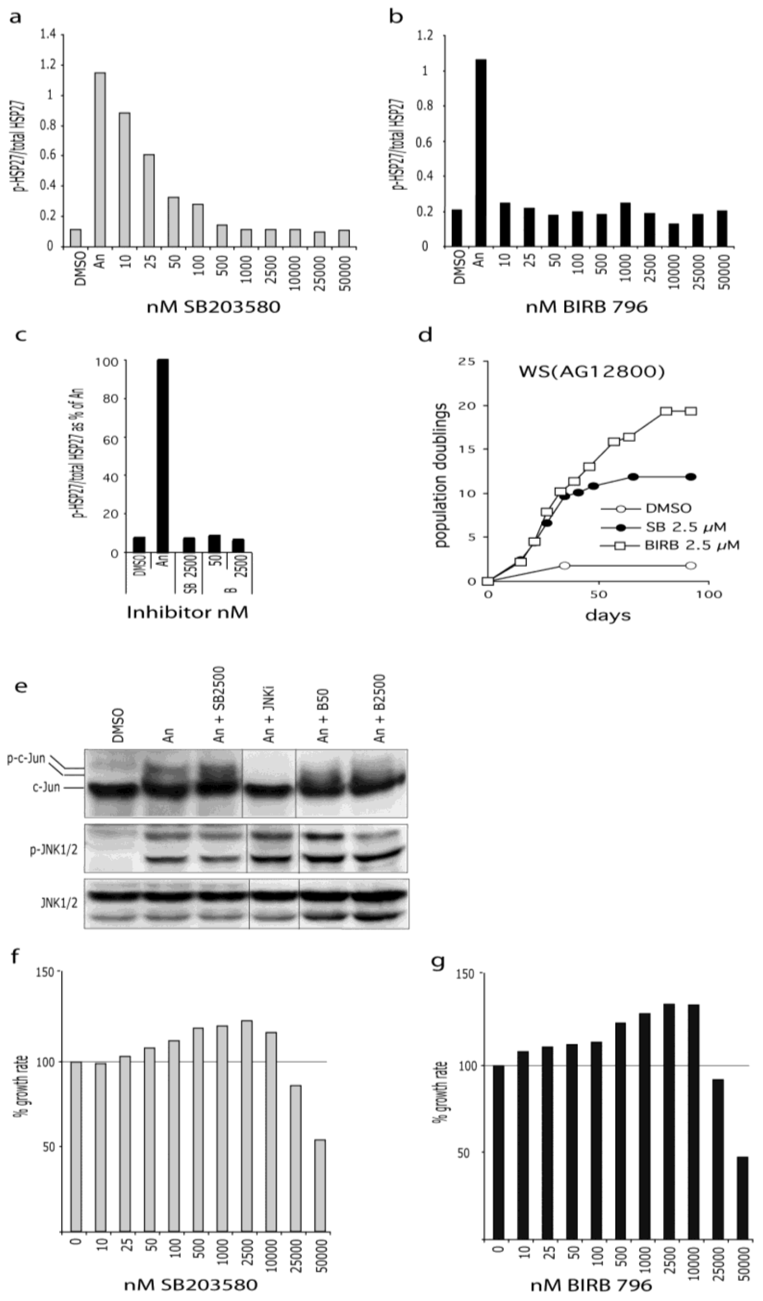

Figure S1. Supplementary Figures. (**a**, **b**) p38 inhibition profiles for SB203580 and BIRB 796 in HCA2^{tert} cells, assays as described in Materials and Methods. The graphs show ratio of pHSP27/HSP27. DMSO is control, An is cells stimulated with 30 μM anisomycin for 45 mins, 10 to 50,000 are cells pre-incubated with inhibitors for2 h prior to anisomycin stimulation. (**c**) Inhibition profiles for SB203580 (SB) and BIRB 796 (B) added to cells 24 h prior to anisomycin stimulation, concentrations given. (**d**) Growth curves for WS(AG12800) cells. (**e**) Immunoblot showing that neither SB203580 nor BIRB 796 significantly inhibit JNK1/2 or prevent their activation at 2.5 μM indicated by the presence of the p-c-Jun doublet in lanes 2,3,5 and 6 in the top panel and the doublet in lanes 2,3,5 and 6 in the middle panel (symbols as for c). Lane 4 shows the effect of a JNK1/2 inhibitor (see reference 37). Note that the sections of each panel are from the same immunoblot and each section has not been handled differently. (**f**, **g**) Effects of SB203580 and BIRB 796 on the growth rates of WS^{tert} cells (repeated experiment): see Figure 1 for details.

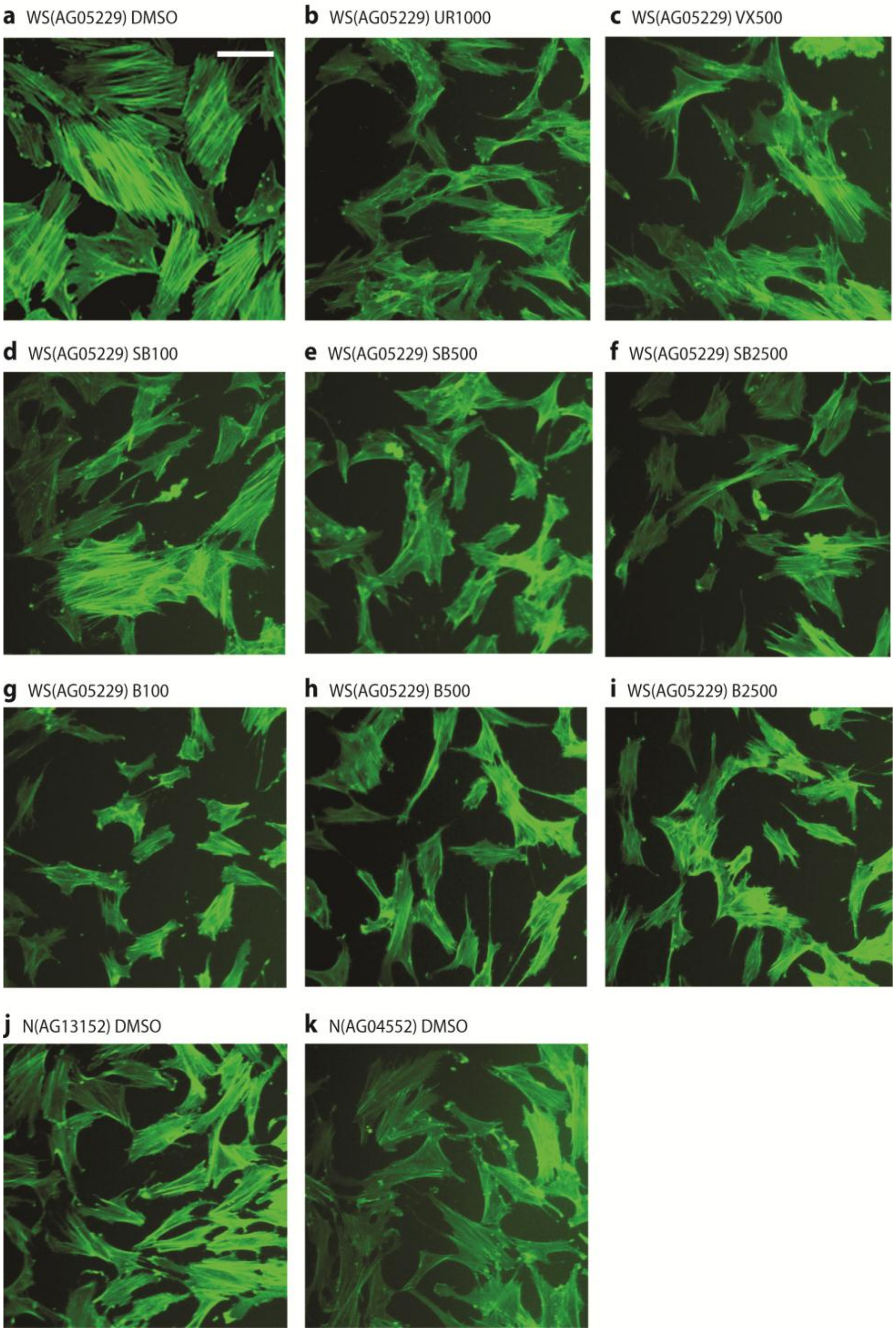

Figure S2. Figure 4. Stress fibre phenotypes of WS(AG05229) cells. Phalloidin stains for WS(AG05229) cells (**a**–**i**) and NDFs (**j**, **k**). Each panel labeled with strain and inhibitor used (symbols for inhibitors as in legend to Figure 3). These are representative figures from many experiments. Bar = 100 μm for each panel.
